# Accuracy of neutrophil lymphocyte ratio for diagnosis of acute appendicitis in children: A diagnostic study

**DOI:** 10.1016/j.amsu.2019.10.013

**Published:** 2019-10-17

**Authors:** Dedi Prasetya, Rochadi, Gunadi

**Affiliations:** a Pediatric Surgery Division, Department of Surgery, Faculty of Medicine, Public Health and Nursing, Universitas Gadjah Mada/Dr. Sardjito Hospital, Yogyakarta, 55281, Indonesia; b PKU Muhammadiyah Wonosobo Hospital, Central Java, 56371, Indonesia

**Keywords:** Accuracy, Acute appendicitis, Children, Diagnosis, Neutrophil lymphocyte ratio

## Abstract

**Background:**

Many biomarkers for diagnosis of acute appendicitis in children have been reported, however, the results are still controversial. We assessed the accuracy of neutrophil-lymphocyte ratio (NLR) for diagnosis of acute appendicitis and discriminating simple and complicated appendicitis in children.

**Methods:**

We included 121 patients with acute appendicitis and 49 children with intussusception as controls who were admitted at our hospital from 2013 to 2017. White blood count (WBC), neutrophil, and NLR were compared between groups.

**Results:**

Neutrophil and NLR were significantly higher in the acute appendicitis group than control (76.17 ± 14.41 *vs.* 62.43 ± 15.9%, *p*=<0.0001; and 8.44 ± 6.63 *vs.* 3.38 ± 2.84, *p*=<0.0001, respectively), while WBC, neutrophil, and NLR were significantly greater in complicated than simple appendicitis (15.86 ± 6.48 *vs.* 12.64 ± 6.27 × 10^3^/μL, *p* = 0,008; 82.64 ± 8.41 *vs.* 68.99 ± 16.23%, *p*=<0.0001; and 11.32 ± 6.87 *vs.* 5.25 ± 4.65, *p*=<0.0001, respectively). The sensitivity, specificity, positive predictive value (PPV), negative predictive value (NPV), area under the receiver operating characteristic (ROC) curve, and cutoff point of NLR for diagnosis of acute appendicitis were 83.5%, 57.7%, 81.4%, 61.2%, 0.764, and 2.87, respectively; whereas the sensitivity, specificity, PPV, NPV, area under the ROC curve, and cutoff point of NLR for differentiating complicated and simple appendicitis were 84.6%, 56.5%, 35.5%, 92.9%, 0.790, and 6.59, respectively.

**Conclusion:**

NLR shows a high accuracy for diagnosis of acute appendicitis and distinguishing a complicated appendicitis from the simple one.

## Highlights

• Many biomarkers for diagnosis of acute appendicitis in children are still controversial.
• Neutrophil lymphocyte ratio (NLR) shows a high accuracy for diagnosis of acute appendicitis.
• NLR reveals a high accuracy for distinguishing a complicated appendicitis from the simple one.

## 1 Introduction

Acute appendicitis is the most common case of acute abdominal pain surgery in children [1]. The mortality is quite high especially when the diagnosis is late, and the rate of late diagnosis of acute appendicitis in children is also quite high between 30 and 65% [2].

Diagnosis of acute appendicitis is determined according to clinical manifestations, laboratory findings, imaging or scoring systems, however, it is sometimes difficult to diagnose an acute appendicitis early. Imaging from computed tomography (CT), which has a high accuracy to diagnose an acute appendicitis, is a relatively expensive, with limited availability and high radiation risks [3].

Currently, there is still no accurate diagnostic method for establishing the diagnosis of acute appendicitis, especially in the early stage. There are several scoring systems used to diagnose an acute appendicitis including: the Alvarado scoring system and pediatric appendicitis score, however, they have several setbacks, such as the score is usually lower in children because the patient is not cooperative or the children is too young to accurately express their complaints. These scoring systems also are unable to distinguish simple appendicitis from the complicated ones [4]. The results of appendicitis scoring system are also still controversial among studies [1].

Many studies have examined the use of biomarkers to establish an early diagnosis of acute appendicitis, such as white blood cell counts (WBC), neutrophil, and neutrophil lymphocyte ratio (NLR) [[5], [6], [7]]. The accuracy of WBC and neutrophil for diagnosis of acute appendicitis varies among studies [1]. These findings might imply the wide variation in pre-test probability among institution where these laboratory tests were conducted [1]. Furthermore, NLR has been shown to have a high accuracy to diagnose acute appendicitis, however, most of the studies were performed on adult subjects and they showed different cutoff values of NLR [6,7]. Therefore, we aimed to investigate the accuracy of NLR for: 1) diagnosis of acute appendicitis; and 2) discriminating simple and complicated appendicitis in children.

## 2 Material and methods

### 2.1 Subjects

This retrospective study consisted of 121 subjects with acute appendicitis and 49 patients with intussusception as controls who were admitted to our hospital from January 2013–December 2017. Data were retrospectively collected from patients’ medical records, including age, gender, symptoms and clinical signs, laboratory and histopathological findings as reference standard. The inclusion criteria were children with acute appendicitis and controls at age of <18 years old, while the exclusion criteria were incomplete medical records. This study was approved by the Ethical Committee of our institution (KE/FK/0118/EC/2018).

The diagnosis of acute appendicitis in our hospital was established according to clinical manifestation, physical examination, laboratory findings and/or abdominal ultrasound. The final diagnosis was determined by histopathological findings.

### 2.2 Neutrophil lymphocyte ratio

Laboratory data were collected including WBC, neutrophil and lymphocyte values. NLR was determined by dividing the neutrophil and lymphocyte values obtained.

### 2.3 Statistical analysis

Data were determined as percentage/frequency and mean ± standard deviation (SD). *t*-test was used to analyze the differences between groups when the data was normally distributed, while Mann-Whitney *U* test was utilized when the data was not normally distributed. By comparing the means of two independent samples, the power of this study was 1.00. The WBC, neutrophil and NRL were evaluated for sensitivity, specificity, positive predictive value (PPV), negative predictive value (NPV), and accuracy. The cutoff value of WBC, neutrophil and NLR for diagnosis of acute appendicitis and distinguishing simple appendicitis from the complicated ones were determined by receiver operating characteristics (ROC) curves.

## 3 Results

### 3.1 Baseline characteristics

Most acute appendicitis patients in our institution were between the ages of 5–10 years old (50%), male (56%) and underwent laparotomy (51.2%). The mean WBC and neutrophil count were 14.33 ± 6.56 × 10^3^/μl and 76.16 ± 14.41%, respectively. Ultrasonography found non-visualized appendix in 23.7% patients, whereas histopathological findings included perforated appendicitis in 51.2% of the children (Table 1).

**Table 1:** Baseline characteristics of children with acute appendicitis.

Characteristics	N (%); mean ± SD (normal range)
Age at surgery (years)	
<5	17 (14)
5-10	60 (49.6)
>10	44 (36.4)
Gender	
Male	68 (56.2)
Female	53 (43.8)
Hemoglobin (g/dL)	12.5 ± 1.8 (10.5–13.5)
WBC (x10^3^/μL)	14.33 ± 6.56 (4–11)
Neutrophil (%)	76.16 ± 14.41 (35–75)
Thrombocyte (x10^3^/μL)	343.2 ± 132.68 (150–400)
CRP (mg/L) (n = 81)	
<5	15 (18.5)
≥5	66 (81.5)
Procalcitonin (μg/L) (n = 69)	
<0.05	10 (14.5)
≥0.05	59 (85.5)
Abdominal ultrasound (n = 59)	
Not visualized	14 (23.7)
Normal	9 (15.3)
Appendicitis	36 (61)
Surgical Approach	
Appendectomy	47 (38.8)
Laparoscopy	12 (10)
Laparotomy	62 (51.2)
Histopathological Finding	
Simple appendicitis	59 (48.8)
Perforated appendicitis	62 (51.2)

CRP, c-reactive protein; SD, standard deviation; WBC, white blood cell.

### 3.2 Accuracy of WBC, neutrophil and neutrophil-lymphocyte ratio for diagnosis of acute appendicitis

Neutrophil and NLR were significantly higher in the acute appendicitis group than control (76.17 ± 14.41 *vs.* 62.43 ± 15.9%, *p*=<0.0001; and 8.44 ± 6.63 *vs.* 3.38 ± 2.84, *p*=<0.0001, respectively), while WBC did not differed between appendicitis acute and control group (*p* = 0.057) (Table 2).

**Table 2:** The accuracy of neutrophil and NLR for diagnosis of acute appendicitis in children.

	Acute appendicitis	Control	*p*	AUC	Cutoff level	Sensitivity (%)	Specificity (%)	PPV (%)	NPV (%)	OR (95% CI)
WBC (x10^3^/μL)	14.33 ± 6.56	12.3 ± 4.29	0.057^#^	N/A	N/A	N/A	N/A	N/A	N/A	N/A
Neutrophil (%)	76.17 ± 14.4	62.43 ± 15.9	<0.0001*	0.756	64.2	83.1	59.2	83.1	59.2	7.11 (3.39–14.9)
NLR	8.44 ± 6.63	3.38 ± 2.84	<0.0001*	0.764	2.87	83.5	57.7	81.4	61.2	6.89 (3.29–14.4)

^#^, *t*-test; *, significant (*p* < 0.05), Mann-Whitney *U* test; WBC, white blood cell; N/A, not determined; NLR, neutrophil-lymphocyte ratio.

**Figure 1:**
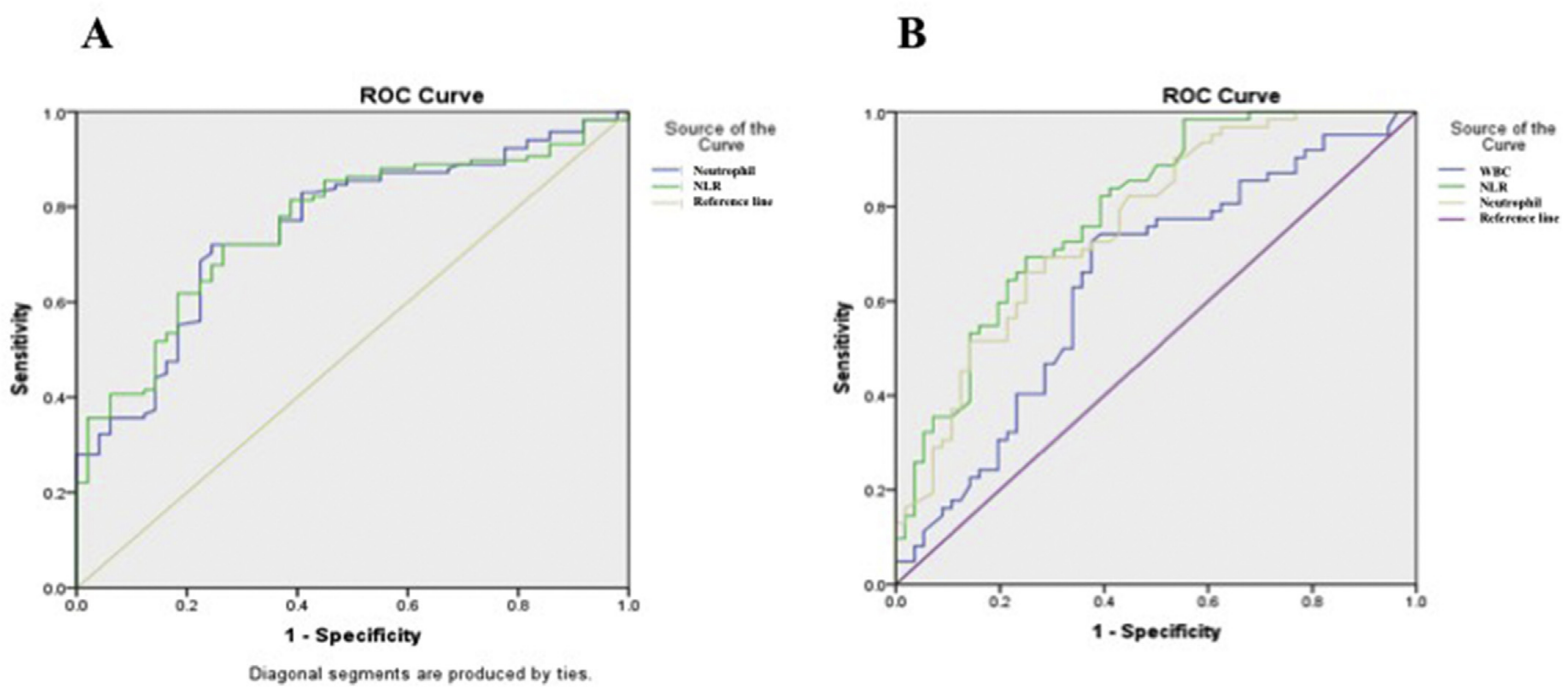
A) ROC curve of neutrophil and NLR for diagnosis of acute appendicitis, with the AUC of 0.756 and 0.764, respectively; B) ROC curve of WBC, neutrophil, and NLR for distinguishing between simple and complicated appendicitis, with the AUC of 0.644, 0.762, and 0.79, respectively.

The sensitivity, specificity, PPV, NPV, area under the ROC curve, and cutoff point of NLR for diagnosis of acute appendicitis were 83.5%, 57.7%, 81.4%, 61.2%, 0.764, and 2.87, respectively, while those of neutrophil were 83.1%, 59.2%, 83.1%, 59.2%, 0.756, and 64.2%, respectively (Fig. 1; Table 2).

### 3.3 Accuracy of WBC, neutrophil and neutrophil-lymphocyte ratio for distinguishing simple and complicated appendicitis

WBC, neutrophil, and NLR were significantly greater in complicated than simple appendicitis (15.86 ± 6.48 *vs.* 12.64 ± 6.27 × 10^3^/μL, *p* = 0,008; 82.64 ± 8.41 *vs.* 68.99 ± 16.23%, *p*=<0.0001; and 11.32 ± 6.87 *vs.* 5.25 ± 4.65, *p*=<0.0001, respectively) (Table 3).

The sensitivity, specificity, PPV, NPV, area under the ROC curve, and cutoff point of NLR, neutrophil, and WBC for differentiating complicated and simple appendicitis were 84.6%, 56.5%, 35.5%, 92.9%, 0.790, and 6.59; 74.5%, 66.7%, 66.1%, 75%, 0.762, and 80.05%; and 66.1%, 62.5%, 66.1%, 62.5%, 0.644 and 13.63, respectively (Fig. 1; Table 3).

**Table 3:** The accuracy of WBC, neutrophil and NLR for distinguishing simple and complicated appendicitis in children.

	Simple appendicitis	Complicated appendicitis	*p*	AUC	Cutoff level	Sensitivity (%)	Specificity (%)	PPV (%)	NPV (%)	OR (95% CI)
WBC (x10^3^/μL)	12.64 ± 6.27	15.86 ± 6.48	0.008*	0.644	13.63	66.1	62.5	66.1	62.5	3.25 (1.53–6.9)
Neutrophil (%)	68.99 ± 16.23	82.64 ± 8.41	<0.0001*	0.762	80.05	74.5	66.7	66.1	75	5.85 (2.62–13.05)
NLR	5.25 ± 4.65	11.32 ± 6.87	<0.0001*	0.79	6.59	84.6	56.5	35.5	92.9	7.15 (2.28–22.4)

*, significant (*p* < 0.05), Mann-Whitney *U* test; WBC, white blood cell; NLR, neutrophil-lymphocyte ratio.

## 4 Discussion

We are able to show that NLR has a high accuracy to diagnose acute appendicitis in children with sensitivity of ~0.84. Our finding is similar with previous studies that showed NLR is a good parameter for diagnosis of acute appendicitis [5,8], although with different cutoff value (our study: 2.87 *vs.* 3.5 [5]). NLR has a better accuracy than WBC or abdominal ultrasound for diagnosis of acute appendicitis [8]. Our study reveals that WBC does not associate with acute appendicitis (Table 2). Furthermore, our patients do not show any abnormality in their appendix (15.3%) and some even have a non-visualized appendix (23.7%) during abdominal ultrasound. It has been known that ultrasound results are very dependent on the operator and have low accuracy for diagnosis of acute appendicitis [7,9].

Our study found that neutrophil is a good variable to diagnose acute appendicitis in children with sensitivity of ~0.83. These results are compatible with previous reports, showing the sensitivity of neutrophil to diagnose acute appendicitis between 0.78 and 0.88 [10,11]. WBC and neutrophil have good sensitivity, however, but low specificity for diagnosis of acute appendicitis [7,12]. We failed to present the association between WBC and diagnosis of acute appendicitis. Moreover, some patients with histopathological findings of appendicitis showed a normal WBC count [12].

We also are able to reveal the capability of NLR to differentiate between simple and complicated appendicitis in children with sensitivity and cutoff value of ~0.85 and 6.59, respectively. Increasing levels of NLR will happen at the early stage of appendix inflammation and continue to increase in 85–95% patients with severe infection process such as in complicated appendicitis [7,13]. These findings were similar to other previous studies, with varying cutoff values between 5 and 8 [6,7]. Furthermore, NLR has been reported as a simple, accurate, and affordable biomarker of subclinical inflammation and simply determined from the differential WBC [14].

It should be noted, this study was a retrospective study, becoming a limitation of study. In addition, there is no single laboratory variable that can be used to diagnose an acute appendicitis precisely [15]. These limitations should be considered during the interpretation of our findings.

Interestingly, we can demonstrate that WBC has a significant impact on distinguishing the simple from the complicated appendicitis (Table 3), with sensitivity and cutoff value of 0.66 and 13.63 × 10^3^/μL, respectively (Table 3). The normal range of WBC varies among studies, with variations in the normal limits of leukocytes used from 9 to 11 × 10^3^/μL with a sensitivity and specificity of 60–80% and 50–100% [12,16]. Although good as a biomarker for diagnosis of acute appendicitis, WBC may not be used to distinguish between simple and complicated appendicitis due to variations in sensitivity and specificity [12].

Moreover, most of our patients are male (56%) with an average age of 5–10 years (50%). These findings were compatible with previous study [17]. Acute appendicitis is more commonly found in adolescents, which might be associated with the large proportion of lymphoid tissue during adolescence, resulting in the appendix being susceptible to obstruction and inflammation [18].

## 5 Conclusions

NLR shows a high accuracy for the diagnosis of acute appendicitis and distinguishing a complicated appendicitis from the simple one.

## Declaration of competing interest

No potential conflict of interest relevant to this article was reported.

## List of Abbreviations

NLR: neutrophil-lymphocyte ratio
NPV: negative predictive value
PPV: positive predictive value
ROC: receiver operating characteristic
WBC: white blood cell count

## Trial registry number

• Name of the registry: Research Registry
• Unique Identifying number or registration ID: researchregistry5082
• Hyperlink to the registration (must be publicly accessible): https://www.researchregistry.com/browse-the-registry#user-researchregistry/registerresearchdetails/5d578081d052c0001018a3ca/
